# Identification of properties important to protein aggregation using feature selection

**DOI:** 10.1186/1471-2105-14-314

**Published:** 2013-10-28

**Authors:** Yaping Fang, Shan Gao, David Tai, C Russell Middaugh, Jianwen Fang

**Affiliations:** 1 College of Science, Institute for Computer Applications, Huazhong Agricultural University, Wuhan 430070, P.R. China; 2 Applied Bioinformatics Laboratory, University of Kansas, 2034 Becker Drive, Lawrence, KS 66047, USA; 3 Department of Pharmaceutical Chemistry, University of Kansas, 2030 Becker Drive, Lawrence, KS 66047, USA; 4 Biometric Research Branch, Division of Cancer Treatment and Diagnosis, National Cancer Institute, 9609 Medical Center Dr, Rockville, MD 20850, USA

**Keywords:** Aggregation, Amyloid, Peptide, Prediction, Feature selection, Machine learning

## Abstract

**Background:**

Protein aggregation is a significant problem in the biopharmaceutical industry (protein drug stability) and is associated medically with over 40 human diseases. Although a number of computational models have been developed for predicting aggregation propensity and identifying aggregation-prone regions in proteins, little systematic research has been done to determine physicochemical properties relevant to aggregation and their relative importance to this important process. Such studies may result in not only accurately predicting peptide aggregation propensities and identifying aggregation prone regions in proteins, but also aid in discovering additional underlying mechanisms governing this process.

**Results:**

We use two feature selection algorithms to identify 16 features, out of a total of 560 physicochemical properties, presumably important to protein aggregation. Two predictors (ProA-SVM and ProA-RF) using selected features are built for predicting peptide aggregation propensity and identifying aggregation prone regions in proteins. Both methods are compared favourably to other state-of-the-art algorithms in cross validation. The identified important properties are fairly consistent with previous studies and bring some new insights into protein and peptide aggregation. One interesting new finding is that aggregation prone peptide sequences have similar properties to signal peptide and signal anchor sequences.

**Conclusions:**

Both predictors are implemented in a freely available web application (http://www.abl.ku.edu/ProA/). We suggest that the quaternary structure of protein aggregates, especially soluble oligomers, may allow the formation of new molecular recognition signals that guide aggregate targeting to specific cellular sites.

## Background

Protein aggregation has been intensely studied experimentally and computationally because the aggregation of protein drugs is of significant concern. It is encountered routinely during the protein refolding, purification, formulation, storage and shipping processes [1,2]. Moreover, it is associated with over 40 human diseases, such as Alzheimer’s, Parkinson, Huntington, prion diseases, and type II diabetes [3]. Interestingly, recently several functional amyloids have been found that play an important role in cellular biology and caused no measurable cytotoxicity [4]. Despite extensive research efforts, the underlying mechanisms of protein aggregation are not completely understood [5]. Many common phenomena related to aggregation, however, have been observed in various experiments [6]. For example, a small segment of one protein can be involved in aggregation while the rest retains a native-like structure. Some short *de novo* peptides can also form fibrils that closely resemble those formed by natural amyloid proteins. Experiments have confirmed many small peptides with lengths as short as 7 [7], 6 [8], 5 [9], and even 4 [10] residues can form aggregates. Swapping an aggregation prone segment from an amyloidogenic protein to a non-amyloidogenic homolog can trigger amyloid formation [11]. These observations suggest that short aggregation prone regions of sequence and structural specificity, rather than full-length sequences, can induce the formation of most if not all aggregates. Although in vivo amyloid disease aggregates may show different characteristics from those formed from protein drugs, as the former are well ordered entities containing cross beta structure fibers while the later are frequently amorphous entities, current prevailing theories consider both amyloid fibers and amorphous aggregates are formed from partially-folded intermediates [12]. Therefore, both amorphous aggregates and fibers may contain similar aggregation prone motifs [13].

A number of computational methods have been developed for predicting peptide aggregation propensities and identifying aggregation prone regions in whole protein sequences [14]. Notable methods include TANGO [15], PAGE [9], FoldAmyloid [16,17], Zyggegator [18], AGGRESCAN [19], and Pafig [20]. In all of these methods, with the exception of Pafig, a small number of physicochemical properties were empirically chosen to develop predictive models. For example, Chiti *et al*. [21] developed an empirical model using hydrophobicity, charge and propensity to predict aggregation rates. The model was later extended to a seven-parameter model designated Zyggegator for predicting absolute aggregation rates [22]. In both models, parameters were fitted using experimental data. Tartaglia *et al*. [3] instead developed a model using physicochemical properties without any free parameters. PAGE includes more properties such as aromatic residue number, parallel or anti-parallel configuration, and accessible surface area [9]. TANGO is a statistical mechanics algorithm for predicting β-aggregation in peptides and proteins. It is based on the assumption that the core regions of an aggregate are fully buried [15]. FoldAmyloid uses packing density which represents the average number of residues within a contact radius of 8.0 Å for 20 amino acid residues obtained from a database consists of protein with sequence identity less than 25% as well as the probability of hydrogen bond formation [23]. It has been demonstrated that regions that possess high packing density can be responsible for amyloidogenic properties. AGGRESCAN is a sequence based aggregation prediction tool based on an aggregation propensity scale for each of the 20 amino acids, which is derived from experimental data [19].

While these methods have generally resulted in good prediction accuracies, little systematic research has been done to determine peptide properties relevant to aggregation and their relative importance. Although Pafig uses machine learning methods to identify features relevant to aggregation, the number of the selected features is very large (41 features). Therefore it is very difficult to evaluate the biological relevance of each selected feature.

The goal of this study is to use feature selection algorithms to identify a small number of features important to protein aggregation. The removal of irrelevant or redundant features often improves the performance of learning models, providing faster and more cost-effective predictions. More importantly, this procedure may provide a better understanding of properties important to aggregation. Therefore, an investigation into physicochemical factors affecting protein aggregation has dual aims, precisely predicting protein aggregation propensities and discovering additional underlying mechanisms governing this important process.

In this study, we initially collect comparatively large training and test datasets from literature. A set of nine classification algorithms are used to test the classifiability of the datasets and define a baseline for performance evaluation of feature selection. We then apply two feature selection methods based on support vector machine (SVM) [24] and Random Forest (RF) [25] algorithms to rank 560 numeric features. SVM and RF classifiers, named ProA-SVM and ProA-RF respectively, with selected features are built, cross-validated and compared to five state-of-the-art methods. To test the reliability of selected features and robustness of the learned models, a leave-one-protein-out (LOPO) cross validation is conducted. Finally, we use our predictors to calculate aggregation propensity profiles for some well-studied proteins to identify aggregation prone regions and compare them to experimental results and other predictive methods.

One interesting finding of this study is that aggregation prone peptide sequences share similar properties with signal peptide and signal anchor sequences. This is supported by our finding that this model for aggregation can be used to accurately distinguish signal peptides from non-signal peptides. A possible relationship between aggregation and localization is also discussed.

## Results and discussion

### Baseline classification of samples using nine algorithms

We first constructed 9 classifiers based on 9 commonly used algorithms and then used these classifiers to establish a performance baseline. The performance of the classifiers was evaluated using the standard 10-fold cross validation. The accuracies of the predictions range from 0.717 (RPART) to 0.797 (GBM) (Table 1). The results show good classifiability on the dataset AP1. Therefore it is feasible to predict peptide propensities using collected features by machine learning methods.

**Table 1:** Performance of 9 classifiers before and after feature selection

	**560 features**		**7 features**		**10 features**	
	**Accuracy**	**MCC**	**Accuracy**	**MCC**	**Accuracy**	**MCC**
SVM-linear	0.759	0.518	0.771	0.542	0.788	0.576
RF	0.748	0.497	0.737	0.474	0.782	0.564
GBM	0.754	0.509	0.718	0.436	0.797	0.593
RPART	0.717	0.435	0.751	0.502	0.729	0.457
NNet	0.754	0.507	0.734	0.468	0.780	0.558
PLS	0.740	0.479	0.788	0.578	0.782	0.565
KNN	0.762	0.530	0.763	0.524	0.763	0.528
NB	0.731	0.465	0.743	0.488	0.790	0.581
Ada	0.754	0.509	0.740	0.479	0.779	0.558

The 7 features are selected by *SVM*-*RFE* and the 10 features are selected by *RF*-*IS*. The 10 fold cross validation of each classifier is conducted on dataset *AP*1.

### Feature selection

**Figure 1:**
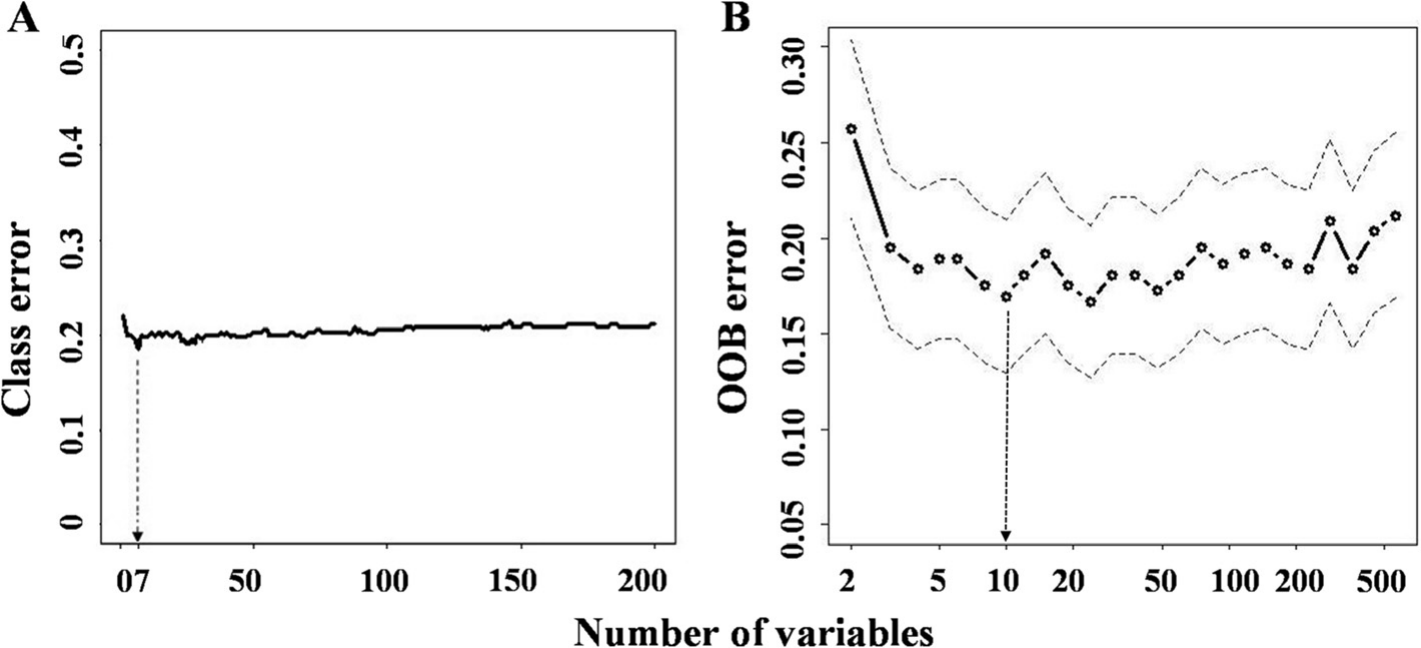
**Dependency of classification performance on the numbers of selected features A) Classification error plotted against the number of feature selected by SVM-RFE, B) OOB error plotted against the number of feature selected by RF-IE.** “Class error” equals to 1 minus classification accuracy, and “OOB error” is the abbreviation of out-of-bag (OOB) error rate which represents error rate for classification.

We use two feature selection methods, namely SVM-RFE and RF-IS, to select features which are important to protein aggregation. The feature selection procedure of both approaches starts with the full set of features and then iteratively eliminates a number or a fraction of the least important features, as determined by the SVM-RFE and RF-IS algorithms. To determine the optimal set of features, the accuracy of the cross validation in each iteration is calculated and plotted against the number of selected features (Figure 1), leading to a selection of the top 7 features by the SVM-RFE method and the top 10 features by the RF-IS method (Table 2). These numbers are chosen because they both represent the first significant minimums with near-best performance while the numbers of features are significantly smaller than the ones with the best performance. For example, in the RF-IS case the best performance is achieved by the model with 24 features. It is clear that the performance gain from the 14 additional features is insignificant from the one with 10 features (Figure 1).

**Table 2:** **Selected top features using SVM**-**RFE and RF**-**IS**

**Method**	*Feature	**Description**
SVM-RFE	ROSM880105	Hydrophilicity of Polar Amino Acid Side-chains
	RICJ880117	Relative preference in alpha helices
	VENT840101	Hydrophobicity, the bitter taste of L-amino acids
	ROBB760110	Conformational properties of middle turn
	PONP800105	Surrounding hydrophobicity in beta-sheet
	ZIMJ680101	Hydrophobicity by statistical methods
	PRAM820103	Shape and surface features of globular proteins
RF-IS	GUYH850101	Partition energy
	VHEG790101	Transfer free energy
	ROSM880105	Hydrophilicity of Polar Amino Acid Side-chains
	CASG920101	Hydrophobicity scale from native protein structures
	PONP800107	Accessibility reduction ratio
	WILM950102	Hydrophobicity coefficient in RP-HPLC
	X15925383	P_agg_
	LEVM780102	Normalized frequency of beta-sheet
	PALJ810111	Normalized frequency of beta-sheet
	PRAM900103	Relative frequency in beta-sheet

*AAIndex database entry numbers.

### Evaluation of feature selection performance

We constructed a group of 9 classifiers using the top 7 features selected by the SVM-RFE method and another group of 9 classifiers with the top 10 features selected by RF-IS to evaluate the efficacy of feature selection. The results of the cross-validation of all classifiers on the dataset AP1 are shown in Table 1. It is observed that, 1) the top 10 features result in improved performance for all 9 tested algorithm; 2) the top 7 features produce enhanced performance of 5 classifiers (SVM, RPART, PLS, KNN and NB), and reduced in the remaining four. Overall, the feature selection process is very effective in removing a significant number of features (> 98%) while improving the performance. In addition, the results imply that selected features are important to the aggregation propensities of those peptides.

We further evaluated the selected 10 and 7 features using LOPO cross-validation. The LOPO results show that SVM and RF achieve good performance (Table 3). Therefore, we applied SVM and RF to build 2 predictors for practical uses: ProA-SVM (Protein Aggregation SVM Predictor) and ProA-RF (Protein Aggregation RF predictor).

**Table 3:** **Results of LOPO cross**-**validation**

**Predictor**	**TP**	**FN**	**FP**	**TN**	**Ac**	**MCC**
ProA-SVM (7 features)	148	36	38	132	0.791	0.5811
ProA-RF (10 features)	146	38	41	129	0.7768	0.5527

*TP*: the number of True Positive samples; *FN*: the number of False Negative samples; *FP*: the number of false positive samples and *TP*: the number of true positive samples. *Ac*: Accuracy. *MCC*: Matthews correlation coefficient.

### Comparison with other methods

**Figure 2:**
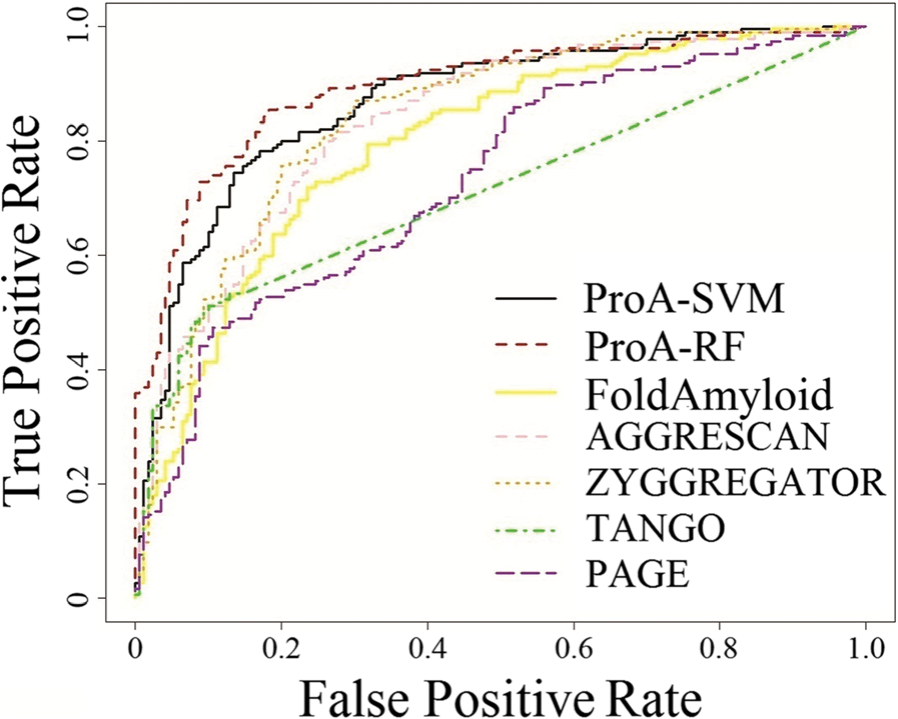
**The receiver operator characteristic (ROC) curves curve for different methods.** Area Under the ROC Curve (AUC): ProA-RF: 0.8929; ProA-SVM: 0.8680, ZYGGREGATOR: 0.8395; AAGRESCAN: 0.8336; FoldAmyloid: 0.7946; PAGE: 0.7303, TANGO: 0.7121.

ProA-SVM and ProA-RF were compared on the dataset AP1 (See Methods) with five state-of-the-art sequence-based predictors, namely AGGRESCAN, TANGO, PAGE, FoldAmyloid, and ZYGGREGATOR. To display the outputs of these predictors together, their values are scaled into the numeric range of 0 to 1 (Figure 2). Considering their different predictive thresholds, the ROC (Receiver Operator Characteristic) curves are used to evaluate the performance of all predictors. Figure 2 clearly shows that the best performance is achieved by ProA-RF with an AUC (Area Under the ROC Curve) of 0.8954, followed by ProA-SVM with 0.8674, ZYGGREGATOR with 0.8395, AAGRESCAN with 0.8336, FoldAmyloid with 0.7946, PAGE with 0.7303, and TANGO with 0.7121. Based on the ROC curve, if the predictive threshold (false positive rate) is set as 0.2, the TN, FN, TP and FP are shown in the following Table 4. It can be seen that the best performance is achieved by ProA-RF with accuracy 0.8192. The followings are ProA-SVM (0.7910), ZYGGREGATOR (0.7768), AAGRESCAN (0.7316), FoldAmyloid (0.7119), TANGO (0.6610) and PAGE (0.6554). Although ProA-RF and ProA-SVM have better or comparable performance to other algorithms, caution should be taken in interpreting the comparison results because of the different data sets and validation strategies used to build these models. The results, nevertheless, clearly demonstrate that the feature selection procedure used in the study is able to identify features important to aggregation.

**Table 4:** Comparison results with other methods

	**TN**	**FN**	**TP**	**FP**	**Negative precision**	**Positive precision**	**Ac**
ProA-RF	136	30	154	34	0.8193	0.8191	0.8192
ProA-SVM	136	40	144	34	0.7727	0.8090	0.7910
ZYGGREGATOR	136	45	139	34	0.7514	0.8035	0.7768
AAGRESCAN	136	61	123	34	0.6904	0.7834	0.7316
FoldAmyloid	136	68	116	34	0.6667	0.7733	0.7119
TANGO	136	86	98	34	0.6126	0.7424	0.6610
PAGE	136	88	96	34	0.6071	0.7385	0.6554

*TP*: the number of True Positive samples; *FN*: the number of False Negative samples; *FP*: the number of false positive samples and *TP*: the number of true positive samples. *Ac*: Accuracy.

### Application to Identification of aggregation prone regions

To compare the ability to identify aggregation prone regions on entire sequences with those of other methods, we use ProA-SVM and ProA-RF to generate aggregation propensity profiles of 37 proteins using sliding windows of length 7. The models are built using the LOPO approach and therefore both predictors scan and predict regions on one protein based on a model built on all other proteins.

**Figure 3:**
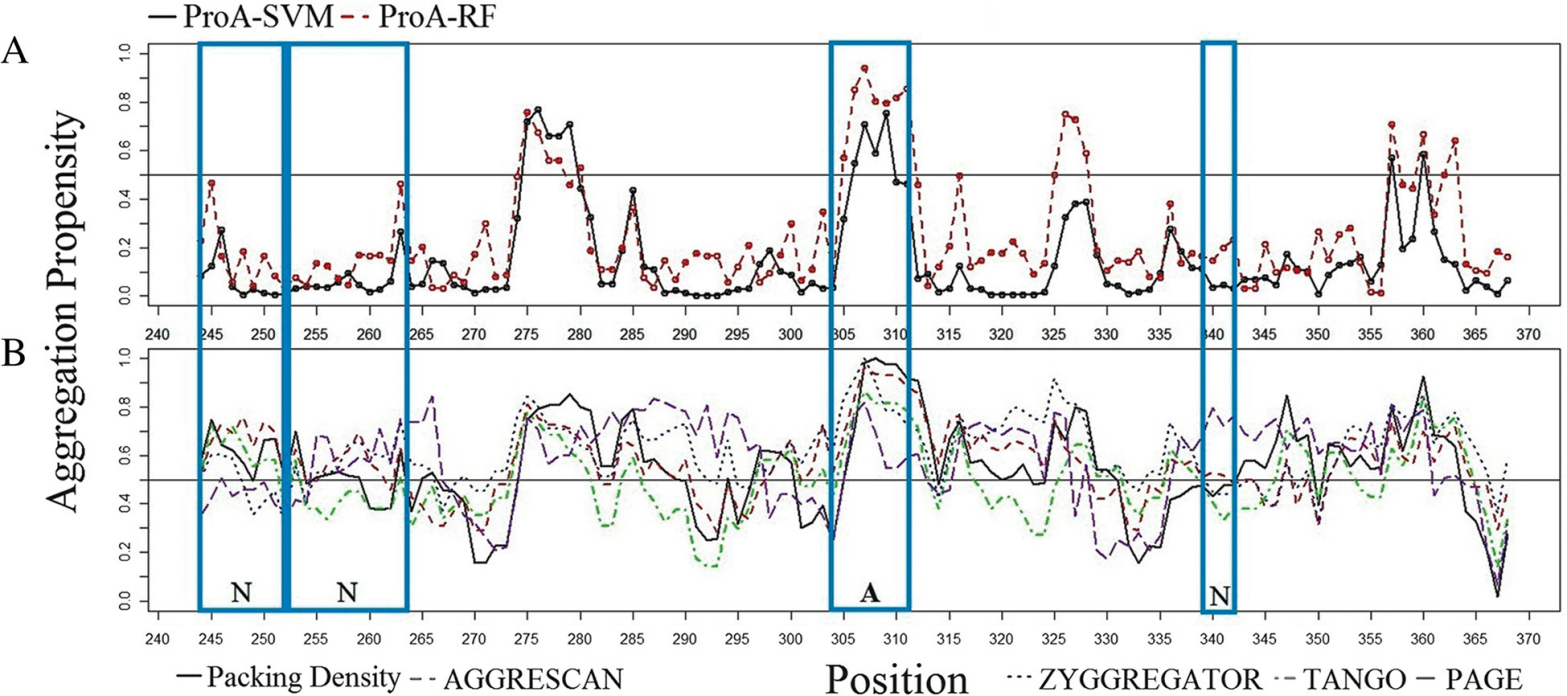
**The predicted aggregation regions of tau protein (region 244–368) by different methods. A**. The predicted aggregation propensity profiles of ProA-SVM (dashed line), ProA-RF (solid line); **B**. The predicted aggregation propensity profiles of ZYGGREGATOR (blue), AAGRESCAN (red), FoldAmyloid (black), PAGE (purple), and TANGO (green). “A” and “N” indicate experimentally confirmed aggregation and non-aggregation regions, respectively.

In most cases, the predictions of all methods are in good agreement with the experiment data. Nevertheless, in some cases the methods developed in this study can identify more true positives and true negatives than other approaches. One example is the aggregation profile of the microtubule-associated protein tau (P10636-8), whose function is involved in promoting microtubule assembly and stability. It has been experimentally validated that tau is involved in Alzheimer’s disease [26]. Figure 3 shows the aggregation propensity profiles by 7 predictors of the region 244–368 in tau. All 7 predictors are able to identify a nonamyloidogenic region ^253^LKNVKSKIGSTE^264^ and an amyloidogenic region ^304^GKVQIVYK^311^. Nevertheless, our methods correctly predict the nonamyloidogenic regions ^244^QTAPVPMPD^252^ and ^339^VKSE^342^, which are predicted as amyloidogenic regions by most of other methods.

### Analysis of selected features

The 16 features from the union of top 7 and top 10 features can be roughly grouped into 5 different categories (Table 2): aggregation propensities (“X15925383”), hydrophobicity (“ROSM880105”, “VENT840101”, “PONP800105”, “ZIMJ680101”,“CASG920101” and “WILM950102”), propensities of secondary structure or conformation (“RICJ880117”, “ROBB760110”, “PRAM900103”, “LEVM780102”, “PALJ810111” and PRAM900103), energy (“GUYH850101” and “VHEG790101”), and accessibility (“PONP800107”). The index “X15925383” calculated using the ZYGGREGATOR method (P_agg_, pH=7) can be decomposed into the properties of hydrophobicity, secondary structure propensities and net charge. Thus these results are consistent with previous findings that hydrophobicity, charges, secondary structure propensities of residues play important roles in the aggregation process. A detailed annotation of selected features is provided in Additional file 1.

### Properties relevant to protein aggregation are important to signal peptides

Among 16 selected features, two are related to protein translocation. “VHEG790101” is the free energy of transferring amino acids from aqueous solution to a lipophilic phase [27], and “PRAM900103” is the statistics of the relative frequency of beta-sheets distributed in signal and nascent peptides [28]. There are 6 features related to hydrophobic properties such as ROSM880105, VENT840101, PONP800105, ZIMJ680101, CASG920101 and WILM950102. It is consistent with previous publications that signal peptide and signal anchor sequences have a distinct hydrophobic region [29].

This observation suggests that peptides with a tendency to aggregate may have similar properties with signal peptides and signal anchors. To test this hypothesis, we downloaded the SignalP version 2.0 dataset (SP) which contains 4 groups of signal peptides and signal anchors [30] (See Methods) and tested them by ProA-SVM and ProA-RF (Tables 4, 5). The accuracy of the predictions by ProA-SVM on the test dataset SP reaches 0.8346 (Table 5), which is even higher than the accuracy of 0.805 obtained in the predictions on the training set AP1. The accuracy of the predictions by ProA-RF (0.77) is also fairly good. Therefore models built on AP1 with selected features can be used to predict signal peptides and signal anchors, indicating aggregation-prone fragments share similar physicochemical properties with these two types of functional sequences.

**Table 5:** **Results of testing ProA**-**SVM and ProA**-**RF on the SP dataset**

**Predictor**	**Dataset**	**TP**	**FN**	**FP**	**TN**	**Ac**	**MCC**
ProA-SVM	EUKSIG.reduc	1022	103	189	936	0.8702	0.7426
	EUKANC.reduc	55	12	10	56	0.8346	0.6695
	GRAM+SIG.reduc	118	51	33	136	0.7515	0.5058
	GRAM-SIG.reduc	286	64	73	277	0.8043	0.6088
	**Total**	**1481**	**230**	**305**	**1405**	**0**.**8436**	**0**.**6879**
ProA-RF	EUKSIG.reduc	896	229	297	828	0.7662	0.5334
	EUKANC.reduc	60	7	9	57	0.8797	0.7597
	GRAM+SIG.reduc	127	42	38	131	0.7633	0.5268
	GRAM-SIG.reduc	290	60	104	246	0.7657	0.5357
	**Total**	**1373**	**338**	**448**	**1262**	**0**.**7702**	**0**.**5416**

*TP*: the number of True Positive samples; *FN*: the number of False Negative samples; *FP*: the number of false positive samples and *TP*: the number of true positive samples. *Ac*, Accuracy; *MCC*, Matthews correlation coefficient.

While it is interesting that aggregation prone peptides share common properties with signal peptides and signal anchors, the results are not particularly surprising since both aggregation and localization processes are associated with inter-molecular hydrophobic interactions. This observation, nevertheless, raises an interesting question whether aggregation prone regions of a protein determine the deposition site of protein aggregates since it has been observed that protein aggregates are deposited at specific cellular sites [31]. We suggest that the quaternary structure of protein aggregates, especially soluble oligomers, may allow the formation of new molecular recognition signals that guide aggregate targeting to specific cellular sites. If this hypothesis is confirmed, it will help us to better understand the molecular basis for protein aggregation and may have significant implications for developing new therapeutic strategies for treating protein aggregation related diseases.

## Methods

### Datasets construction

We compiled a set of peptides known to form or not form aggregates determined experimentally from the literature. After removing ambiguous entries, we obtained a set of 354 samples (peptides) including 184 positives and 170 negatives. 297 samples originated from 37 proteins (the similarity is below 25%) but 57 of them are *de novo* peptides. The average lengths of positives and negatives are 12.72 and 12.6 residues while the corresponding standard deviations are 8.23 and 5.53, respectively. Considering the confusion of protein names and sequences in the literature, we referred these peptide sequences to the UniProtKB/Swiss-Prot database and used their UniProtKB/Swiss-Prot IDs as their unique identifiers wherever possible. All entries of Aggregation Propensity dataset 1 (AP1) and their original references are provided in Additional file 2.

We obtained signal peptide data (SP) from the SignalP version 2.0, which was previously used for identifying prokaryotic and eukaryotic signal peptides and predicting their cleavage sites [32]. SP contains 4 non-redundant datasets in which signal peptides and signal anchors are designated as positive samples and others as negative samples:

1. EUKSIG.reduc: 2250 Eukaryotes signal peptides (1125 positives and 1125 negatives);

2. EUKANC.reduc: 133 Eukaryotes signal anchors (67 positives and 66 negatives);

3. GRAM+SIG.reduc: 338 Gram-positive bacteria signal peptides (169 positives and 169 negatives).

4. GRAM-SIG.reduc: 700 Gram-negative bacteria signal peptides (350 positives and 350 negatives).

### Feature extraction and peptide encoding

We compiled a collection of 560 features including 544 physicochemical properties obtained from AAindex database [33] and 16 additional features collected from published literatures (Additional file 3). All features were scaled into a range between 0 and 1. Each peptide is encoded by an array of 560 features, each of which is the average of corresponding features of the amino acid residues in the peptide. Thus, the *i*^*th*^ sample peptide is represented by 560 features in a form of ${\vec{x}}_{i}=\left(x_{i1},x_{i2,}\ldots x_{\mathrm{ij}}\ldots x_{i560}\right)$. If a peptide or a protein contains an amino acid residue with ‘NA’ value for a particular physicochemical property in AAindex, this amino acid are not used to calculate the average value for this physicochemical property.

### Classification algorithms

We use nine different classification algorithms to establish a performance baseline for classification and to test the efficacy of feature selection. These 9 algorithms include: SVMs [24], RF [25], Generalized Boosted Models (GBM) [34], Recursive Partitioning And Regression Trees (RPART) [35], Neural Network (NNet) [36], Partial Least Squares (PLS) [37], K Nearest Neighbours (KNN) [38], Naive Bayes (NB) [39], and Ada Boost (Ada) [40]. These algorithms are implemented in R using the caret package [41] in this study. The following is a brief description of these algorithms. Details can be found in the manual of the caret package and references cited therein. The specific commands including parameter tuning of each method used in the study can be found in the Additional file 4.

The SVM algorithm is a supervised learning method, which can be used for classification or regression. It derives parameters of the maximum-margin to construct an optimized separating hyperplane by solving the optimization. The fit of SVM classifiers includes the selection of kernel, the kernel's parameters, and soft margin parameter C.

The Random Forest algorithm is an ensemble machine learning method that utilizes many independent decision trees to perform classification or regression. Each of the member trees is built on bootstrap samples from the training data by a random subset of available variables. Each Random Forest model built in this study consists of 500 decision trees. The number of variable randomly sampled in each tree is , where M is the number of total variables.

GBM integrates the concept of boosting methods with that of classification or regression trees. Using GBM for classification is to build a sequence of simple decision trees, where each successive tree is for the prediction residuals of the preceding tree. A learned function must be chosen from a restricted class of functions to most closely approximate the gradient of the loss function.

RPART is a method used to create decision trees and iteratively split the data on each of the nodes using user-specified splitting criteria. The algorithm chooses the split that partitions the data into separate nodes such that it minimizes the sum of the squared deviations of the mean in all of those nodes. The process continues until it satisfies a user-specified stopping criterion.

NNet is a computational model that imitates the structure and function of biological neural networks. It is typically defined by three types of parameters: the interconnection pattern between neurons, the learning process for updating the weights of the interconnections, and the activation function. The NNet used in this study is a feed-forward neural network with single hidden layer.

PLS is an extension of the multiple linear regression model, bearing some relation to principal component analysis. A PLS model is acquired by projecting the predicted variables and the observable variables into a new space. The PLS can be used for classification and regression.

KNN is one of the simplest machine learning algorithms. In KNN classification, a sample is assigned to the class with most common amongst its *k* nearest neighbors (*k* is a positive integer) by a majority vote of its neighbors. Despite its simplicity, KNN is often shown good performance comparable to other state-of-the-art algorithms.

NB uses the Bayes rule to compute the posterior probability of a categorical class variable, with the conditional independence assumption. One advantage of NB is that the decoupling of the class conditional feature distributions in calculation alleviates the problem known as the “curse of dimensionality.” Another advantage is that NB does not need to accurately estimate the absolute accuracy of each class because the classification outcome is determined by the relative probabilities of all classes.

Ada is an adaptive algorithm that can be used to build a series of classifiers, where subsequent classifiers are adapted in favor of those instances misclassified by previous classifiers. Although Ada can be sensitive to noisy data and statistics outliers, it has been found that it is often less susceptible to the overfitting problem than many other learning algorithms.

### SVM-RFE and RF-IS

In this study, feature selection is executed using two state-of-the-art algorithms, SVM based recursive feature elimination (SVM-RFE) [42] and a Random Forest importance spectrum based feature selection algorithm (RF-IS) [43]. SVM-RFE is based on a backward sequential selection, which starts with all the features and removes one feature each time. The removed feature is the one whose removal minimizes the variation of weights. In this work, SVM-RFE uses linear kernel and C type SVMs [24].

RF-IS eliminates iteratively a number or a fraction of the least important features. Random Forest uses two methods to measure the importance, including the mean decrease in accuracy and mean decrease in node impurity measured by the Gini index. The second approach is used in this study and 20% fraction of features (default values in R package varSelRF ) [43] is removed in each loop. The features are selected in this approach using the command varSelRF(trainX, trainY, c.sd = 1, mtryFactor = 1, ntree = 500, ntreeIterat = 200, vars.drop.num = NULL, vars.drop.frac = 0.2, whole.range = TRUE, recompute.var.imp = TRUE, verbose = FALSE, returnFirstForest = TRUE, fitted.rf = NULL).

### Cross validation

We use the standard 10-fold and Leave-One-Protein-Out (LOPO) cross validation methods to estimate the performance of classifiers. In 10-fold cross validation, we randomly split the AP1 into ten equal portions. We use nine portions as the training dataset and the remaining one as the test dataset. All parameters are fitted using an “inner” 10-fold cross validation in order to avoid potential overfitting problem. The procedure is repeated nine more times until each portion is used as the testing dataset once. LOPO mimics real-world applications by grouping all of the peptides (samples) according to the proteins to which they belong. We obtain 37 protein groups and a non-protein group comprising all *de novo* peptides. In LOPO cross validation, the test dataset consists of samples from one protein or the non-protein group, with all other samples included in the training dataset.

### Performance comparison to other methods

We compare our methods with five state-of-the-art sequence-based methods including TANGO [15], PAGE [9], FoldAmyloid [23], Zyggregator [18], and AGGRESCAN [19]. To visualize and compare the results of those methods, outputs from these methods are scaled into a range of [0, 1]. Since different methods may use different classification thresholds, the area under the receiver operator characteristic (ROC) curve (AUC) is used to evaluate the performances of all methods. AUC is considered as a robust metric for classifier evaluation and comparison. An ROC curve is generated by varying the output threshold of a classifier and plotting the true positive rate (sensitivity) against the false positive rate (1 – specificity) for each threshold value. The ROC curve has been widely used in many protein aggregation studies as a standard threshold-independent metric [19,23]. We also provide the Matthews correlation coefficient (MCC) [44]for each method:

$$\mathrm{MCC}=\frac{\mathrm{TP}\times \mathrm{TN}-\mathrm{FP}\times \mathrm{FN}}{\sqrt{\left(\mathrm{TP}+\mathrm{FP}\right)\left(\mathrm{TP}+\mathrm{FN}\right)\left(\mathrm{TN}+\mathrm{FP}\right)\left(\mathrm{TN}+\mathrm{FN}\right)}}$$

Where TP is the number of true positives, TN the number of true negatives, FP the number of false positives and FN the number of false negatives. MCC is in the range between −1 and +1. A MCC of +1 represents a perfect prediction, 0 an average random prediction and −1 an inverse prediction.

### Prediction of peptide aggregation propensities and calculation of aggregation propensity profile of the whole sequences

In this study we aim to develop models for predicting aggregation propensity of short peptides (i.e. 3–25 amino acid residues) and aggregation propensity profiles of longer peptides (> 25 amino acid residues) or entire proteins. The overall propensity of longer peptides or whole proteins is less significant since short aggregation prone regions, rather than the full-length sequences, are probably responsible for inducing the formation of most if not all aggregation.

Each short peptide is encoded using an input vector composed of selected features. For a longer peptide or complete protein sequence, a sliding symmetrical local window centered at a particular amino acid residue is used to scan the sequence. Each local window is also encoded with an input vector. The input vector is then used by the predictive models to calculate the aggregation propensity of the short peptide or local window. The predicted value, scaled to a range from 0 to 1, is assigned to the short peptide or the central residue of the window. The predicted values for all of the local windows from the N-terminus to the C-terminus provide an aggregation propensity profile for longer peptides or whole protein sequences. The short peptides or the regions with values above the threshold (default is 0.5) are considered as aggregation prone peptides or regions.

For predicting aggregation propensity profiles, we set the default window size to 7 amino acid residues. Using a shorter window may result in a profile with poor smoothness and a longer window may contain more than 2 or more aggregation prone regions [15]. In addition, experimental measurements and theoretical calculations have indicated that approximate 7 residues are required to span the distance of protofilaments [45]. Furthermore, the optimal window length in the FoldAmyloid algorithm was found to be 7 amino acid residues [23]. Based on these considerations, a window size of 7 amino acid residues is chosen as the default size.

## Conclusion

In this study, 16 physicochemical properties have been identified important to protein aggregation. These findings confirm that hydrophobicity, secondary structure propensities and net charge play important roles in protein aggregation. Two sequence-based predictors (ProA-SVM and ProA-RF) are built to predict peptide aggregation propensities based on the SVMs and RF algorithms. Both predictors demonstrate good generalization abilities and can be used to identify aggregation prone regions of proteins. An interesting new finding is that aggregation peptides have similar properties to those of signal peptides and anchors, which indicates that the aggregation prone regions may also determine the deposition location of protein aggregates. We suggest that the quaternary structure of protein aggregates may allow the formation of neo- signals that guide aggregate targeting to specific cellular sites. If this hypothesis is confirmed, it will provide better understanding of the molecular basis for protein aggregation, and may have significant implications for developing new therapeutic strategies for treating protein aggregation related diseases.

## Abbreviations

ProA-SVM: Protein Aggregation SVM Predictor; ProA-RF: Protein Aggregation RF predictor; SVM: Support Vector Machine; RF: Random Forest; SVM-RFE: SVM based recursive feature elimination; RF-IS: Random Forest importance spectrum based feature selection; LOPO: leave-one-protein-out; GBM: Generalized Boosted Model; RPART: Recursive Partitioning And Regression Tree; NNet: Neural Network; PLS: Partial Least Square; KNN: K-Nearest Neighbour; NB: Naive Bayes; TP: The number of True Positive samples; FN: The number of False Negatives samples; FP: The number of False Positives samples; TN: The number of True Negatives samples; Ac: Accuracy; MCC: Matthews correlation coefficient.

## Competing interests

The authors declare that they have no competing interests.

## Authors’ contributions

JF conceived the project. SG and YF carried out the study with input from CM and JF. YF, SG, CM and JF drafted the manuscript. All authors read and approved the final manuscript.

## Supplementary Material

Additional file 1 Selected features and their AAindex records. Click here for file

Additional file 2 The dataset AP1 (Aggregation Propensity 1). Click here for file

Additional file 3 16 additional features collected from published literatures. Click here for file

Additional file 4 Commands of the classification algorithms used in the study. Click here for file
