# Supplementary material for: Identification of properties important to protein aggregation using feature selection
Source: BMC Bioinformatics. 2013 Oct 28;14:314. doi: 10.1186/1471-2105-14-314 (PMC3819749; doi:10.1186/1471-2105-14-314)
Supplement: Additional file 1 — Selected features and their AAindex records. [file 1471-2105-14-314-S1.doc]

## Supplementary Material 1: Selected features and their AAindex records

| **7 top numeric features selected by SVM-RFE** | **10 top numeric features selected by RF-IS** |
| --- | --- |
| ROSM880105* | GUYH850101 |
| RICJ880117 | VHEG790101 |
| VENT840101 | ROSM880105* |
| ROBB760110 | CASG920101 |
| PONP800105 | PONP800107 |
| ZIMJ680101 | WILM950102 |
| PRAM820103 | X15925383 |
|  | LEVM780102 |
|  | PALJ810111 |
|  | PRAM900103 |

* are common features by two methods.

The AAindex description of those features were list as below:

Each entry was started by “//”

//

H ROSM880105

D Hydropathies of amino acid side chains, pi-values in pH 7.0 (Roseman, 1988)

R PMID:3398047

A Roseman, M.A.

T Hydrophilicity of Polar Amino Acid Side-chains is Markedly Reduced by

Flanking Peptide Bonds

J J. Mol. Biol. 200, 513-522 (1988) (Pro missing)

I A/L R/K N/M D/F C/P Q/S E/T G/W H/Y I/V

0.39 -3.95 -1.91 -3.81 0.25 -1.30 -2.91 0.00 -0.64 1.82

1.82 -2.77 0.96 2.27 NA -1.24 -1.00 2.13 1.47 1.30

//

H RICJ880117

D Relative preference value at C" (Richardson-Richardson, 1988)

R LIT:1408116 PMID:3381086

A Richardson, J.S. and Richardson, D.C.

T Amino acid preferences for specific locations at the ends of alpha helices

J Science 240, 1648-1652 (1988)

I A/L R/K N/M D/F C/P Q/S E/T G/W H/Y I/V

0.7 1.1 1.5 1.4 0.4 1.1 0.7 0.6 1. 0.7

0.5 1.3 0. 1.2 1.5 0.9 2.1 2.7 0.5 1.

//

H VENT840101

D Bitterness (Venanzi, 1984)

R LIT:1103107b PMID:6521488

A Venanzi, T.J.

T Hydrophobicity parameters and the bitter taste of L-amino acids

J J. Theor. Biol. 111, 447-450 (1984)

I A/L R/K N/M D/F C/P Q/S E/T G/W H/Y I/V

0. 0. 0. 0. 0. 0. 0. 0. 0. 1.

1. 0. 0. 1. 0. 0. 0. 1. 1. 1.

//

H ROBB760110

D Information measure for middle turn (Robson-Suzuki, 1976)

R PMID:1003471

A Robson, B. and Suzuki, E.

T Conformational properties of amino acid residues in globular proteins

J J. Mol. Biol. 107, 327-356 (1976)

C ROBB760108 0.960 ROBB760113 0.957 BEGF750103 0.903

CRAJ730103 0.887 PALJ810106 0.864 CHOP780101 0.863

TANS770110 0.805 CHAM830101 0.804

I A/L R/K N/M D/F C/P Q/S E/T G/W H/Y I/V

-4.7 2.0 3.9 1.9 6.2 -2.0 -4.2 5.7 -2.6 -7.0

-6.2 2.8 -4.8 -3.7 3.6 2.1 0.6 3.3 3.8 -6.2

//

H PONP800105

D Surrounding hydrophobicity in beta-sheet (Ponnuswamy et al., 1980)

R LIT:0608056 PMID:7397216

A Ponnuswamy, P.K., Prabhakaran, M. and Manavalan, P.

T Hydrophobic packing and spatial arrangement of amino acid residues in

globular proteins

J Biochim. Biophys. Acta 623, 301-316 (1980)

I A/L R/K N/M D/F C/P Q/S E/T G/W H/Y I/V

14.60 13.24 11.79 13.78 15.90 12.02 13.59 14.18 15.35 14.10

16.49 13.28 16.23 14.18 14.10 13.36 14.50 13.90 14.76 16.30

//

H ZIMJ680101

D Hydrophobicity (Zimmerman et al., 1968)

R LIT:2004109b PMID:5700434

A Zimmerman, J.M., Eliezer, N. and Simha, R.

T The characterization of amino acid sequences in proteins by statistical

methods

J J. Theor. Biol. 21, 170-201 (1968)

I A/L R/K N/M D/F C/P Q/S E/T G/W H/Y I/V

0.83 0.83 0.09 0.64 1.48 0.00 0.65 0.10 1.10 3.07

2.52 1.60 1.40 2.75 2.70 0.14 0.54 0.31 2.97 1.79

//

H PRAM820103

D Correlation coefficient in regression analysis (Prabhakaran-Ponnuswamy, 1982)

R LIT:2004113b

A Prabhakaran, M. and Ponnuswamy, P.K.

T Shape and surface features of globular proteins

J Macromolecules 15, 314-320 (1982) Regression analysis of solvent contact area

and spatial position

I A/L R/K N/M D/F C/P Q/S E/T G/W H/Y I/V

0.687 0.590 0.489 0.632 0.263 0.527 0.669 0.670 0.594 0.564

0.541 0.407 0.328 0.577 0.600 0.692 0.713 0.632 0.495 0.529

//

H GUYH850101

D Partition energy (Guy, 1985)

R LIT:2004051b PMID:3978191

A Guy, H.R.

T Amino acid side-chain partition energies and distribution of residues in

soluble proteins

J Biophys. J. 47, 61-70 (1985)

I A/L R/K N/M D/F C/P Q/S E/T G/W H/Y I/V

0.10 1.91 0.48 0.78 -1.42 0.95 0.83 0.33 -0.50 -1.13

-1.18 1.40 -1.59 -2.12 0.73 0.52 0.07 -0.51 -0.21 -1.27

//

H VHEG790101

D Transfer free energy to lipophilic phase (von Heijne-Blomberg, 1979)

R LIT:0509382 PMID:477664

A von Heijne, G. and Blomberg, C.

T Trans-membrane translocation of proteins: The direct transfer model

J Eur. J. Biochem. 97, 175-181 (1979)

I A/L R/K N/M D/F C/P Q/S E/T G/W H/Y I/V

-12.04 39.23 4.25 23.22 3.95 2.16 16.81 -7.85 6.28 -18.32

-17.79 9.71 -8.86 -21.98 5.82 -1.54 -4.15 -16.19 -1.51 -16.22

//

H CASG920101

D Hydrophobicity scale from native protein structures (Casari-Sippl, 1992)

R PMID:1569551

A Casari, G. and Sippl, M.

T Structure-derived Hydrophobic Potential. Hydrophobic Potential Derived from

X-ray Structures of Globular Proteins is able to Identify Native Folds

J J. Mol. Biol. 224, 725-732 (1992)

I A/L R/K N/M D/F C/P Q/S E/T G/W H/Y I/V

0.2 -0.7 -0.5 -1.4 1.9 -1.1 -1.3 -0.1 0.4 1.4

0.5 -1.6 0.5 1.0 -1.0 -0.7 -0.4 1.6 0.5 0.7

//

H PONP800107

D Accessibility reduction ratio (Ponnuswamy et al., 1980)

R LIT:0608056 PMID:7397216

A Ponnuswamy, P.K., Prabhakaran, M. and Manavalan, P.

T Hydrophobic packing and spatial arrangement of amino acid residues in

globular proteins

J Biochim. Biophys. Acta 623, 301-316 (1980)

I A/L R/K N/M D/F C/P Q/S E/T G/W H/Y I/V

3.70 2.53 2.12 2.60 3.03 2.70 3.30 3.13 3.57 7.69

5.88 1.79 5.21 6.60 2.12 2.43 2.60 6.25 3.03 7.14

//

H WILM950102

D Hydrophobicity coefficient in RP-HPLC, C8 with 0.1%TFA/MeCN/H2O (Wilce et al.

1995)

R

A Wilce, M.C., Aguilar, M.I. and Hearn, M.T.

T Physicochemical basis of amino acid hydrophobicity scales: evaluation of four

new scales of amino acid hydrophobicity coefficients derived from RP-HPLC of

peptides

J Anal Chem. 67, 1210-1219 (1995)

I A/L R/K N/M D/F C/P Q/S E/T G/W H/Y I/V

2.62 1.26 -1.27 -2.84 0.73 -1.69 -0.45 -1.15 -0.74 4.38

6.57 -2.78 -3.12 9.14 -0.12 -1.39 1.81 5.91 1.39 2.30

//

X15925383

ZYGGREGATOR

//

H LEVM780102

D Normalized frequency of beta-sheet, with weights (Levitt, 1978)

R LIT:0411042 PMID:708713

A Levitt, M.

T Conformational preferences of amino acids in globular proteins

J Biochemistry 17, 4277-4285 (1978)

I A/L R/K N/M D/F C/P Q/S E/T G/W H/Y I/V

0.90 0.99 0.76 0.72 0.74 0.80 0.75 0.92 1.08 1.45

1.02 0.77 0.97 1.32 0.64 0.95 1.21 1.14 1.25 1.49

//

H PALJ810111

D Normalized frequency of beta-sheet in alpha+beta class (Palau et al., 1981)

R LIT:0805095 PMID:7118409

A Palau, J., Argos, P. and Puigdomenech, P.

T Protein secondary structure

J Int. J. Peptide Protein Res. 19, 394-401 (1981) LG :a set of protein samples

formed by 44 proteins. CF :a set of protein samples formed by 33 proteins.

I A/L R/K N/M D/F C/P Q/S E/T G/W H/Y I/V

0.82 0.99 1.27 0.98 0.71 1.01 0.54 0.94 1.26 1.67

0.94 0.73 1.30 1.56 0.69 0.65 0.98 1.25 1.26 1.22

//

H PRAM900103

D Relative frequency in beta-sheet (Prabhakaran, 1990)

R LIT:1614053b PMID:2390062

A Prabhakaran, M.

T The distribution of physical, chemical and conformational properties in

signal and nascent peptides

J Biochem. J. 269, 691-696 (1990) Original reference of these three data:

Creighton, T.E. In "Protein Structure and Melecular Properties", (Freeman,

W.H., ed.), San Francisco P.235 (1983)

I A/L R/K N/M D/F C/P Q/S E/T G/W H/Y I/V

0.90 0.99 0.76 0.72 0.74 0.80 0.75 0.92 1.08 1.45

1.02 0.77 0.97 1.32 0.64 0.95 1.21 1.14 1.25 1.49
