# Supplementary material for: Identification of properties important to protein aggregation using feature selection
Source: BMC Bioinformatics. 2013 Oct 28;14:314. doi: 10.1186/1471-2105-14-314 (PMC3819749; doi:10.1186/1471-2105-14-314)
Supplement: Additional file 2 — The dataset AP1 (Aggregation Propensity 1). [file 1471-2105-14-314-S2.doc]

## Supplementary Material 2: The dataset AP1 (Aggregation Propensity 1)

ProteinID: Swiss-Prot accession number; Label: 1 means positive and -1 means negative.

| **Peptide**  **ID** | **Protein**  **ID** | **Sequence** | **Label** | **Starting**  **Position** | **End**  **Position** | **Ref** |
| --- | --- | --- | --- | --- | --- | --- |
| 1 | P10636-8 | PGGGKVQIVYKPV | 1 | 301 | 313 |  |
| 2 | P10636-8 | PGGGKVYKPV | -1 | 301 | 313 |  |
| 3 | P10636-8 | PGGGKNAEVYKPV | -1 | 301 | 313 |  |
| 4 | P10636-8 | PGGGKVQIVEKPV | -1 | 301 | 313 |  |
| 5 | P10636-8 | QTAPVPMPDLKNVKSKIGSTENLKHQPGGGKVQIVY | -1 | 244 | 310 |  |
| 6 | P10636-8 | KPVDLSKVTSKCGSLGNIHHKPGGGQVEVKSEKLDF | -1 | 311 | 346 |  |
| 7 | P10636-8 | KDRVQSKIGSLDNITHVPGGGN | -1 | 347 | 368 |  |
| 8 | P10636-8 | QTAPVPMPDLKNVKSKIGSTE | -1 | 244 | 264 |  |
| 9 | P10636-8 | NLKHQPGGGKVQIVYKPVDLSKVTSKCGSLGNIHHKPGGGQVE | 1 | 265 | 338 |  |
| 10 | P10636-8 | VKSE | -1 | 339 | 342 |  |
| 11 | P10636-8 | KLDFKDRVQSKIGSLDNITHVPGGGN | -1 | 343 | 368 |  |
| 12 | P10636-8 | QTAPVPMPD | -1 | 244 | 252 |  |
| 13 | P10636-8 | LKNVKSKIGSTE | -1 | 253 | 264 |  |
| 14 | P10636-8 | NLKHQPGGGKVQIVYKPVD | 1 | 265 | 314 |  |
| 15 | P10636-8 | LSKVTSKCGSLGNIHHKPGGGQVE | -1 | 315 | 338 |  |
| 16 | P10636-8 | VKSEKLDFKDRVQSKIGSLDNITHVPGGGN | -1 | 339 | 368 |  |
| 17 | P10636-8 | GKVQIVYK | 1 | 304 | 311 |  |
| 18 | P10636-8 | VQIVYK | 1 | 306 | 311 |  |
| 19 | P10636-8 | VDLSKVTSK | -1 | 313 | 321 |  |
| 20 | P10636-8 | VTSKCGSLGNIHHKPGGG | -1 | 318 | 335 |  |
| 21 | P10636-8 | GQVEVSKE | -1 | 335 | 342 |  |
| 22 | P05067 | DAEFRHDSGYEVHHQKLVFFAEDVGSNKGAIIGLMVGGVV | 1 | 672 | 711 |  |
| 23 | P05067 | VPHQKLVFFAEDVGS | 1 | 683 | 697 |  |
| 24 | P05067 | VHPQKLVFFAEDVGS | 1 | 683 | 697 |  |
| 25 | P05067 | VHHPKLVFFAEDVGS | 1 | 683 | 697 |  |
| 26 | P05067 | VHHQPLVFFAEDVGS | 1 | 683 | 697 |  |
| 27 | P05067 | KKPVFFAED | -1 | 686 | 694 |  |
| 28 | P05067 | KKLPFFAED | -1 | 686 | 694 |  |
| 29 | P05067 | KKLVPFAED | -1 | 686 | 694 |  |
| 30 | P05067 | VHHQKLVPFAEDVGS | -1 | 683 | 697 |  |
| 31 | P05067 | KKLVFPAED | -1 | 686 | 694 |  |
| 32 | P05067 | KKLVFFPED | 1 | 686 | 694 |  |
| 33 | P05067 | VHHQKLVFFAPDVGS | -1 | 683 | 697 |  |
| 34 | P05067 | VHHQKLVFFAEPVGS | 1 | 683 | 697 |  |
| 35 | P05067 | VHHQKLVFFAEDPGS | 1 | 683 | 697 |  |
| 36 | P05067 | VHHQKLVFFAEDVPS | 1 | 683 | 697 |  |
| 37 | P05067 | KKLVFFAED | 1 | 686 | 694 |  |
| 38 | P05067 | VHHQKLVFFAEDVGS | 1 | 683 | 697 |  |
| 39 | P05067 | KLVFF | -1 | 687 | 691 |  |
| 40 | P05067 | QKLVFFA | -1 | 686 | 692 |  |
| 41 | P05067 | HQKLVFFAE | -1 | 685 | 693 |  |
| 42 | P05067 | HHQKLVFFAED | 1 | 684 | 694 |  |
| 43 | P05067 | VHHQKLVFFAEDV | 1 | 683 | 695 |  |
| 44 | P05067 | EVHHQKLVFFAEDVG | 1 | 682 | 696 |  |
| 45 | P05067 | YEVHHQKLVFFAEDVGS | 1 | 681 | 697 |  |
| 46 | P05067 | GYEVHHQKLVFFAEDVGSN | 1 | 680 | 698 |  |
| 47 | P05067 | SGYEVHHQKLVFFAEDVGSNK | 1 | 679 | 699 |  |
| 48 | P05067 | DSGYEVHHQKLVFFAEDVGSNKG | 1 | 678 | 700 |  |
| 49 | P05067 | HDSGYEVHHQKLVFFAEDVGSNKGA | 1 | 677 | 701 |  |
| 50 | P37840 | EQVTNVGGAVVTGVTAVA | 1 | 61 | 78 |  |
| 51 | P37840 | TVNGVGEVTATAVQGVAV | 1 | 64 | 84 |  |
| 52 | P37840 | VTNVGGAVVTGVTAVA | 1 | 63 | 78 |  |
| 53 | P37840 | EQVTNVGGAVVTG | 1 | 61 | 73 |  |
| 54 | P37840 | VGGAVVTGV | 1 | 66 | 74 |  |
| 55 | P14621 | STAQSLKSVDYEVFGRV | -1 | 2 | 18 |  |
| 56 | P14621 | QGVSFRMYTEDEARKI | -1 | 19 | 34 |  |
| 57 | P14621 | GVVGWVKNTSKGTVTGQVQG | 1 | 35 | 54 |  |
| 58 | P14621 | PEDKVNSMKSWLSKV | -1 | 55 | 69 |  |
| 59 | P14621 | GSPSSRIDRTNFSNEKT | -1 | 70 | 86 |  |
| 60 | P14621 | ISKLEYSNFSVRY | 1 | 87 | 99 |  |
| 61 | P61769 | IQRTPKIQVYSRHPAE | -1 | 21 | 36 |  |
| 62 | P61769 | NGKSNFLNCYVSG | -1 | 37 | 49 |  |
| 63 | P61769 | FHPSDIEVDLLK | -1 | 50 | 61 |  |
| 64 | P61769 | NGERIEKVEHSDLSFSKD | -1 | 62 | 79 |  |
| 65 | P61769 | DWSFYLLYYTEFT | 1 | 79 | 91 |  |
| 66 | P61769 | DWSFYLLYYTEFTPTGKDEYA | 1 | 79 | 99 |  |
| 67 | P61769 | PTGKDEYACRVNHVT | -1 | 92 | 106 |  |
| 68 | P61769 | LSQPKIVKWDRDM | -1 | 107 | 119 |  |
| 69 | P61769 | RTPKIQVYSR | -1 | 23 | 32 |  |
| 70 | P61769 | QVYSRHPAEN | -1 | 28 | 37 |  |
| 71 | P61769 | HPAENGKSNF | -1 | 33 | 42 |  |
| 72 | P61769 | LNCYVSGFHP | -1 | 43 | 52 |  |
| 73 | P61769 | SGFHPSDIEV | -1 | 48 | 57 |  |
| 74 | P61769 | SDIEVDLLKN | -1 | 53 | 62 |  |
| 75 | P61769 | DLLKNGERIE | -1 | 58 | 67 |  |
| 76 | P61769 | GERIEKVEHS | -1 | 63 | 72 |  |
| 77 | P61769 | KVEHSDLSFS | -1 | 68 | 77 |  |
| 78 | P61769 | DLSFSKDWSF | 1 | 73 | 82 |  |
| 79 | P61769 | KDWSFYLLYY | 1 | 78 | 87 |  |
| 80 | P61769 | YLLYYTEFTP | 1 | 83 | 92 |  |
| 81 | P61769 | TEFTPTEKDE | 1 | 88 | 97 |  |
| 82 | P61769 | TEKDEYACRV | -1 | 93 | 102 |  |
| 83 | P61769 | YACRVNHVTL | -1 | 98 | 107 |  |
| 84 | P61769 | NHVTLSQPKI | -1 | 103 | 112 |  |
| 85 | P03036 | MQTLSERLKKRRIALKY | -1 | 1 | 17 |  |
| 86 | P03036 | YKMTQTELATKAGVK | -1 | 15 | 29 |  |
| 87 | P03036 | YKQQSIQLIEAGVTKR | -1 | 28 | 43 |  |
| 88 | P03036 | TKRPRFLYEIAMALNSD | 1 | 41 | 57 |  |
| 89 | P03036 | AMALNCDPVWLQYGTKRGKA | -1 | 51 | 70 |  |
| 90 | P02185 | VLSEGEWQLVLHVWAKVEA | 1 | 2 | 20 |  |
| 91 | P02185 | EGEWQLVLHVWAKVEADVAGHGQDILIRLFK | 1 | 5 | 35 |  |
| 92 | P02185 | DVAGHGQDILIRLFKS | 1 | 21 | 36 |  |
| 93 | P02185 | KSHPET | -1 | 35 | 40 |  |
| 94 | P02185 | HPETLEKFDRFKHLK | -1 | 37 | 51 |  |
| 95 | P02185 | TEAEMKA | -1 | 52 | 58 |  |
| 96 | P02185 | SEDLKKHGVTVLTALGAILK | -1 | 59 | 78 |  |
| 97 | P02185 | KKGHHEAE | -1 | 79 | 86 |  |
| 98 | P02185 | ELKPLAQSHA | -1 | 86 | 95 |  |
| 99 | P02185 | ATKHKIP | -1 | 95 | 101 |  |
| 100 | P02247 | GWEIPEPYVWDESFRVFY | -1 | 1 | 18 |  |
| 101 | P02247 | GTDFKYKGKL | -1 | 109 | 118 |  |
| 102 | P02247 | YEQLDEEHKKIFKGIFDCIRD | -1 | 18 | 38 |  |
| 103 | P02247 | RDNSA | -1 | 37 | 41 |  |
| 104 | P02247 | SAPNLATLVKVTTNHFTHEEAMMD | 1 | 40 | 63 |  |
| 105 | P02247 | DAAKYSEV | -1 | 63 | 70 |  |
| 106 | P02247 | EVVPHKKMHKDFLEKIGGL | 1 | 69 | 87 |  |
| 107 | P02247 | GLSAPVD | -1 | 86 | 92 |  |
| 108 | P02247 | AKNVDYCKEWLVNHIK | -1 | 93 | 108 |  |
| 109 | P00287 | LEVLLGSG | -1 | 1 | 8 |  |
| 110 | P00287 | LEVLLGSGDGSLVFV | 1 | 1 | 15 |  |
| 111 | P00287 | SGDGSL | -1 | 7 | 12 |  |
| 112 | P00287 | SLVFVPSEFS | -1 | 11 | 20 |  |
| 113 | P00287 | SEFSV | -1 | 17 | 21 |  |
| 114 | P00287 | SEFSVPSGEK | -1 | 17 | 26 |  |
| 115 | P00287 | KIVFKNNA | -1 | 26 | 33 |  |
| 116 | P00287 | GEKIVFKNNAGFPHNVVFDE | 1 | 24 | 43 |  |
| 117 | P00287 | KIVFKNNAGFPH | -1 | 26 | 37 |  |
| 118 | P00287 | KNNAGFPHNV | -1 | 30 | 39 |  |
| 119 | P00287 | PHNVVFDEDEIP | -1 | 36 | 47 |  |
| 120 | P00287 | IPAGVDAVKISM | 1 | 46 | 57 |  |
| 121 | P00287 | EIPAGV | -1 | 45 | 50 |  |
| 122 | P00287 | DAVKIS | -1 | 51 | 56 |  |
| 123 | P00287 | MPEEELL | -1 | 57 | 63 |  |
| 124 | P00287 | MPEEELLNAPGETYVVTL | 1 | 57 | 74 |  |
| 125 | P00287 | ELLNAPGETY | -1 | 61 | 70 |  |
| 126 | P00287 | NAPGETY | -1 | 64 | 70 |  |
| 127 | P00287 | APGET | -1 | 65 | 69 |  |
| 128 | P00287 | GETYVVTL | 1 | 67 | 74 |  |
| 129 | P00287 | ETYVVT | -1 | 68 | 73 |  |
| 130 | P00287 | VTLDTKGTY | -1 | 72 | 80 |  |
| 131 | P00287 | GTYSFYT | 1 | 78 | 84 |  |
| 132 | P00287 | TYSFYC | -1 | 79 | 84 |  |
| 133 | P00287 | YTSPHQGAGMV | -1 | 83 | 93 |  |
| 134 | P00287 | MVGKVTVN | -1 | 92 | 99 |  |
| 135 | P00287 | GTVSFVTSPHQGAGMVGKVTVN | 1 | 78 | 99 |  |
| 136 | P00974 | RPDFSLEPPYTGPSK | -1 | 36 | 50 |  |
| 137 | P00974 | LSQTFVYGGSRAKRNN | 1 | 64 | 79 |  |
| 138 | P00974 | PSKARIIRY | -1 | 48 | 56 |  |
| 139 | P00974 | KRNNFKSAEDS | -1 | 76 | 86 |  |
| 140 | P00974 | ARIIRYFYNAKAG | -1 | 51 | 63 |  |
| 141 | P00974 | FKSAEDSMRTSGGA | -1 | 80 | 93 |  |
| 142 | P00974 | NAKAGLSQT | -1 | 59 | 67 |  |
| 143 | A4ITV1 | MKVIFLKDVKG | 1 | 1 | 11 |  |
| 144 | A4ITV1 | KGKKGEIKNVAD | -1 | 12 | 23 |  |
| 145 | A4ITV1 | GYANNFLFKQG | 1 | 24 | 34 |  |
| 146 | A4ITV1 | LAIEATPA | -1 | 35 | 42 |  |
| 147 | A4ITV1 | TPANLKALEAQKQKEQR | -1 | 40 | 56 |  |
| 148 | P80031 | DQKEAALVDMVNDGVEDLRCKYATLIYT | -1 | 80 | 107 |  |
| 149 | P80031 | YEAGKEKYVKELPEHLKPFETLLSQ | -1 | 109 | 133 |  |
| 150 | P80031 | QISFADYNLLDLLRIHQVLN | 1 | 145 | 164 |  |
| 151 | P80031 | PLLSAYVARLSA | -1 | 172 | 183 |  |
| 152 | P80031 | PKIKAFLA | -1 | 185 | 192 |  |
| 153 | Q13813 | AYVKKLDSGTGKELVLAL | -1 | 967 | 975 |  |
| 154 | Q13813 | YDYQEKSPREVTMKKGD | -1 | 976 | 992 |  |
| 155 | Q13813 | DILTLLNSTNKDWWKVEVND | 1 | 992 | 1011 |  |
| 156 | Q13813 | GGKDWWKVGG | -1 | 1002 | 1007 |  |
| 157 | Q13813 | DWWKVEVNDRQGFVPA | 1 | 1003 | 1018 |  |
| 158 | Q13813 | DILTLLNSTNKDWWKVEVNDRQGFVPA | 1 | 992 | 1018 |  |
| 159 | P48052 | VPSNEEQIKNLLQLEAQEHLQY | -1 | 31 | 52 |  |
| 160 | P48052 | VPSNEEQIKKLLELEAKKHLQY | -1 | 31 | 52 |  |
| 161 | P48052 | FVNVQAVKVFLESQGIAY | 1 | 71 | 88 |  |
| 162 | P48052 | FVNVEAVKAFLEAHGIAY | 1 | 71 | 88 |  |
| 163 | P19892 | AVGKSNLLSRYARNEFSA | -1 | 19 | 39 |  |
| 164 | P19892 | RFRAVTSAYYRGAVG | -1 | 73 | 87 |  |
| 165 | P19892 | TRRTTFESVGRWLDELKIHSD | -1 | 95 | 115 |  |
| 166 | P19892 | AVSVEEGKALAEEEGLF | -1 | 134 | 150 |  |
| 167 | P19892 | STNVKTAFEMVILDIYNNV | 1 | 159 | 177 |  |
| 168 | P14204 | DHPAVMEGTKTILETDSNLS | -1 | 9 | 28 |  |
| 169 | P14204 | EPSEQFIKQHDFSSY | -1 | 35 | 49 |  |
| 170 | P14204 | VNGMELSKQILQENPH | -1 | 62 | 77 |  |
| 171 | P14204 | EVEDYFEEAIRAGLH | -1 | 87 | 101 |  |
| 172 | P14204 | TESKEKITQYIYHVLNGEIL | 1 | 107 | 126 |  |
| 173 | P0A2D5 | DFSTMRRIVRNLLKELGYN | -1 | 13 | 31 |  |
| 174 | P0A2D5 | EDGVDALNKLQAGGY | -1 | 37 | 51 |  |
| 175 | P0A2D5 | MDGLELLKTIRADSAY | -1 | 63 | 78 |  |
| 176 | P0A2D5 | AKKENIIAAAQAGASGY | 1 | 90 | 106 |  |
| 177 | P0A2D5 | PFTAATLEEKLNKIFEKLGMY | 1 | 110 | 130 |  |
| 178 | P00322 | GTGNTEKMAELIAKGIIESGKDY | -1 | 8 | 30 |  |
| 179 | P00322 | EESEFEPFIEEISTKISY | -1 | 62 | 79 |  |
| 180 | P00322 | GDGKWMRDFEQRMNGYGSV | -1 | 91 | 109 |  |
| 181 | P00322 | EPDEAEQDSIEFGKKIANIY | -1 | 119 | 139 |  |
| 182 | P20171 | GVGKSALTIQLIQNHFVY | 1 | 13 | 30 |  |
| 183 | P20171 | EYSAMRDQYMRTGEG | -1 | 63 | 77 |  |
| 184 | P20171 | INNTKSFEDIHQYREQIKRVKDS | -1 | 84 | 106 |  |
| 185 | P20171 | ARTVESRQAQDLARSYGIP | -1 | 122 | 140 |  |
| 186 | P20171 | RQGVEDAFYTLVREIRQHK | 1 | 149 | 167 |  |
| 187 | Q51912 | VTIKANLIFANGFTQTAEFKG | 1 | 113 | 133 |  |
| 188 | Q51912 | KGTFEKATSEAYAYADTLKKDNGEY | 1 | 132 | 156 |  |
| 189 | Q51912 | GEYTVDVADKGYTLNIKFAGD | 1 | 154 | 174 |  |
| 190 | P19909 | TYKLINGKTLKGETTTEA | -1 | 373 | 391 |  |
| 191 | P19909 | GDAATAEKVFKQYANDNGVD | -1 | 392 | 411 |  |
| 192 | P19909 | GEWTYDDATKTFTVTE | 1 | 412 | 427 |  |
| 193 | P04156 | QGGGTHSQWN | -1 | 91 | 100 |  |
| 194 | P04156 | HSQWNKPSKP | -1 | 96 | 105 |  |
| 195 | P04156 | KPSKPKTNMK | -1 | 101 | 110 |  |
| 196 | P04156 | KTNMKHMAGA | -1 | 106 | 115 |  |
| 197 | P04156 | HMAGAAAAGA | -1 | 111 | 120 |  |
| 198 | P04156 | AAAGAVVGGL | -1 | 116 | 125 |  |
| 199 | P04156 | VVGGLGGYML | -1 | 121 | 130 |  |
| 200 | P04156 | GGYMLGSAMS | -1 | 126 | 135 |  |
| 201 | P04156 | GSAMSRPIIH | -1 | 131 | 140 |  |
| 202 | P04156 | FGSDYEDRYY | -1 | 141 | 150 |  |
| 203 | P04156 | EDRYYRENMH | -1 | 146 | 155 |  |
| 204 | P04156 | RENMHRYPNQ | -1 | 151 | 160 |  |
| 205 | P04156 | RYPNQVYYRP | -1 | 156 | 165 |  |
| 206 | P04156 | VYYRPMDEYS | -1 | 161 | 170 |  |
| 207 | P04156 | MDEYSNQNNF | -1 | 166 | 175 |  |
| 208 | P04156 | NQNNFVHDCV | -1 | 171 | 180 |  |
| 209 | P04156 | VHDCVNITIK | 1 | 176 | 185 |  |
| 210 | P04156 | NITIKQHTVT | -1 | 181 | 190 |  |
| 211 | P04156 | QHTVTTTTKG | -1 | 186 | 195 |  |
| 212 | P04156 | TTTKGENFTE | -1 | 191 | 200 |  |
| 213 | P04156 | ENFTETDVKM | -1 | 196 | 205 |  |
| 214 | P04156 | TDVKMMERVV | -1 | 201 | 210 |  |
| 215 | P04156 | MERVVEQMCI | -1 | 206 | 215 |  |
| 216 | P04156 | EQMCITQYER | -1 | 211 | 220 |  |
| 217 | P04156 | TQYERESQAY | -1 | 216 | 225 |  |
| 218 | P04156 | ESQAYYQRGS | -1 | 221 | 230 |  |
| 219 | P04156 | YQRGSSMVLF | -1 | 226 | 235 |  |
| 220 | P04156 | SMVLFSSPPV | 1 | 231 | 240 |  |
| 221 | P04156 | SSPPVILLIS | 1 | 236 | 245 |  |
| 222 | P04156 | ILLISFLIFL | 1 | 241 | 250 |  |
| 223 | P04156 | FLIFLIVG | 1 | 246 | 253 |  |
| 224 | P61626 | RCELARTLKR | 1 | 23 | 32 |  |
| 225 | P61626 | RTLKRLGMDG | -1 | 28 | 37 |  |
| 226 | P61626 | LGMDGYRGIS | -1 | 33 | 42 |  |
| 227 | P61626 | YRGISLANWM | -1 | 38 | 47 |  |
| 228 | P61626 | LANWMCLAKW | 1 | 43 | 52 |  |
| 229 | P61626 | CLAKWESGYN | -1 | 48 | 57 |  |
| 230 | P61626 | ESGYNTRATN | -1 | 53 | 62 |  |
| 231 | P61626 | TRATNYNAGD | -1 | 58 | 67 |  |
| 232 | P61626 | YNAGDRSTDY | -1 | 63 | 72 |  |
| 233 | P61626 | RSTDYGIFQI | -1 | 68 | 77 |  |
| 234 | P61626 | GIFQINSRYW | -1 | 73 | 82 |  |
| 235 | P61626 | NSRYWCNDGK | -1 | 78 | 87 |  |
| 236 | P61626 | CNDGKTPGAV | -1 | 83 | 92 |  |
| 237 | P61626 | TPGAVNACHL | -1 | 88 | 97 |  |
| 238 | P61626 | NACHLSCSAL | -1 | 93 | 102 |  |
| 239 | P61626 | LQDNIADAVA | -1 | 103 | 112 |  |
| 240 | P61626 | ADAVACAKRV | -1 | 108 | 117 |  |
| 241 | P61626 | CAKRVVRDPQ | -1 | 113 | 122 |  |
| 242 | P61626 | VRDPQGIRAW | -1 | 118 | 127 |  |
| 243 | P61626 | GIRAWVAWRN | -1 | 123 | 132 |  |
| 244 | P61626 | VAWRNRCQNR | -1 | 128 | 137 |  |
| 245 | P61626 | RCQNRDVRQY | -1 | 133 | 142 |  |
| 246 | P61626 | DVRQYVQGCG | -1 | 138 | 147 |  |
| 247 | De novo | YTVIIE | 1 |  |  |  |
| 248 | De novo | WTVIIE | 1 |  |  |  |
| 249 | De novo | VTVIIE | 1 |  |  |  |
| 250 | De novo | TTVIIE | 1 |  |  |  |
| 251 | De novo | SYVIIE | 1 |  |  |  |
| 252 | De novo | SVVIIE | 1 |  |  |  |
| 253 | De novo | STVYIE | 1 |  |  |  |
| 254 | De novo | STVWIE | 1 |  |  |  |
| 255 | De novo | STVTIE | 1 |  |  |  |
| 256 | De novo | STVNIE | 1 |  |  |  |
| 257 | De novo | STVLIE | 1 |  |  |  |
| 258 | De novo | STVIYE | 1 |  |  |  |
| 259 | De novo | STVIIY | 1 |  |  |  |
| 260 | De novo | STVIIW | 1 |  |  |  |
| 261 | De novo | STVIIV | 1 |  |  |  |
| 262 | De novo | STVIIT | 1 |  |  |  |
| 263 | De novo | STVIIS | 1 |  |  |  |
| 264 | De novo | STVIIQ | 1 |  |  |  |
| 265 | De novo | STVIIN | 1 |  |  |  |
| 266 | De novo | STVIIM | 1 |  |  |  |
| 267 | De novo | STVIIL | 1 |  |  |  |
| 268 | De novo | STVIII | 1 |  |  |  |
| 269 | De novo | STVIIF | 1 |  |  |  |
| 270 | De novo | STVIIE | 1 |  |  |  |
| 271 | De novo | STVIID | 1 |  |  |  |
| 272 | De novo | STVIIA | 1 |  |  |  |
| 273 | De novo | STVIFE | 1 |  |  |  |
| 274 | De novo | STVFIE | 1 |  |  |  |
| 275 | De novo | STVEIE | 1 |  |  |  |
| 276 | De novo | STSIIE | 1 |  |  |  |
| 277 | De novo | STQIIE | 1 |  |  |  |
| 278 | De novo | STNIIE | 1 |  |  |  |
| 279 | De novo | STLIIE | 1 |  |  |  |
| 280 | De novo | STFIIE | 1 |  |  |  |
| 281 | De novo | STEIIE | 1 |  |  |  |
| 282 | De novo | SSVIIE | 1 |  |  |  |
| 283 | De novo | SQVIIE | 1 |  |  |  |
| 284 | De novo | SNVIIE | 1 |  |  |  |
| 285 | De novo | SMVIIE | 1 |  |  |  |
| 286 | De novo | SLVIIE | 1 |  |  |  |
| 287 | De novo | SIVIIE | 1 |  |  |  |
| 288 | De novo | SGVIIE | 1 |  |  |  |
| 289 | De novo | SFVIIE | 1 |  |  |  |
| 290 | De novo | SEVIIE | 1 |  |  |  |
| 291 | De novo | SDVIIE | 1 |  |  |  |
| 292 | De novo | SAVIIE | 1 |  |  |  |
| 293 | De novo | QTVIIE | 1 |  |  |  |
| 294 | De novo | NTVIIE | 1 |  |  |  |
| 295 | De novo | MTVIIE | 1 |  |  |  |
| 296 | De novo | LTVIIE | 1 |  |  |  |
| 297 | De novo | ITVIIE | 1 |  |  |  |
| 298 | De novo | GTVIIE | 1 |  |  |  |
| 299 | De novo | FTVIIE | 1 |  |  |  |
| 300 | De novo | ETVIIE | 1 |  |  |  |
| 301 | De novo | DTVIIE | 1 |  |  |  |
| 302 | De novo | ATVIIE | 1 |  |  |  |
| 303 | P61769 | NHVTLS | 1 | 103 | 108 |  |
| 304 | P61769 | LLYYTE | 1 | 84 | 89 |  |
| 305 | P61769 | KIVKWD | 1 | 111 | 116 |  |
| 306 | P61769 | KDWSFY | 1 | 78 | 83 |  |
| 307 | P61769 | FYLLYY | 1 | 82 | 87 |  |
| 308 | Q17217 | VEALYL | 1 | 36 | 41 |  |
| 309 | Q17217 | LYQLEN | 1 | 102 | 107 |  |
| 310 | Q17217 | LVEALY | 1 | 35 | 40 |  |
| 311 | P10997 | NFGAIL | 1 | 55 | 60 |  |
| 312 | P10997 | FLVHSS | 1 | 48 | 53 |  |
| 313 | P61769 | SNFLNCYVSGFHPSDIEVDLLK | 1 | 40 | 61 |  |
| 314 | P61769 | NHVTLSQ | 1 | 103 | 109 |  |
| 315 | P10997 | NFGAILSS | 1 | 55 | 62 |  |
| 316 | P10997 | AFGAILSS | 1 | 55 | 62 |  |
| 317 | P10997 | NFAAILSS | 1 | 55 | 62 |  |
| 318 | P10997 | NFGAALSS | 1 | 55 | 62 |  |
| 319 | P10997 | NFGAIASS | 1 | 55 | 62 |  |
| 320 | P10997 | TNVGSNTY | 1 | 63 | 70 |  |
| 321 | P10997 | QRLANFLVH | 1 | 43 | 51 |  |
| 322 | P10997 | SNNFGAIL | 1 | 53 | 60 |  |
| 323 | P10997 | NFLVHSSNN | 1 | 47 | 55 |  |
| 324 | P05067 | KPFTARFEGRIFSRSDELRALITEITGE | 1 | 364 | 391 |  |
| 325 | P05067 | KPFLARVEGRIFSRSDELRAYITAYTGE | 1 | 364 | 391 |  |
| 326 | P05067 | KPFTARISGRLFSRSDELKTIIATITGE | 1 | 364 | 391 |  |
| 327 | P05067 | KPYIARFEGRLFSRSDELRAVIEAHTGE | 1 | 364 | 391 |  |
| 328 | P05067 | KPFIARFEGRLFSRSDELKAIIKELTGE | 1 | 364 | 391 |  |
| 329 | P05067 | KPFLARFRGRIFSRSDELRTLIAAFTGE | 1 | 364 | 391 |  |
| 330 | P05067 | DAEFRHDSGYEVHHQKLVFFAEDVGSNKGAIIGLMVGGVVIA | 1 | 672 | 713 |  |
| 331 | P37840 | VTGVTAVQKTV | 1 | 71 | 82 |  |
| 332 | P02766 | CPLMVKVLDAV | 1 | 30 | 40 |  |
| 333 | P02766 | YTIAALLSPYS | 1 | 125 | 135 |  |
| 334 | P23370 | RVEKVAILGLMVLA | 1 | 36 | 49 |  |
| 335 | P06396 | SFNNGDCFILD | 1 | 209 | 219 |  |
| 336 | P02788 | NAGDVAFV | 1 | 556 | 563 |  |
| 337 | P02788 | NFGSVQFV | 1 | 556 | 563 |  |
| 338 | P02735 | SFFSFLGEAFD | 1 | 20 | 30 |  |
| 339 | P04156 | DCVNITIKQHTVTT | 1 | 178 | 191 |  |
| 340 | P04156 | DIKIMERVVEQMCTTQY | 1 | 202 | 218 |  |
| 341 | P04156 | AGAAAAGAVVGGLGG | 1 | 113 | 127 |  |
| 342 | P04156 | MKHMAGAAAAGAVV | 1 | 109 | 122 |  |
| 343 | P05453 | PQGGYQQYN | 1 | 75 | 83 |  |
| 344 | P05453 | GNNQQNY | 1 | 7 | 13 |  |
| 345 | P22303 | AEFHRWSSYMVYWK | 1 | 586 | 599 |  |
| 346 | Q9Y287 | EASNCFAIRHFENKFAVETLICSRTVKKNIIEEN | 1 | 244 | 266 |  |
| 347 | Q9Y287 | EASNCFAIRHFENKFAVETLICFNLFLNSQEKHY | 1 | 244 | 266 |  |
| 348 | De novo | VTVKVNAVKVTV | 1 |  |  |  |
| 349 | P01308 | KETAAAKFERQHMDSSTSAA | 1 | 27 | 46 |  |
| 350 | P02185 | IKYLEFISQAIIHVLHSR | 1 | 102 | 119 |  |
| 351 | Q9NY65 | MLSNTTAIAEAWARL | 1 | 377 | 391 |  |
| 352 | P04350 | GYLTVAAVFR | 1 | 308 | 318 |  |
| 353 | P08825 | SYGGEGIGNVAVAGELPVAGKTAVAGRVPIIGAVGFGGPAGAAGAVSIAGR | 1 | 66 | 116 |  |
| 354 | P08825 | GNLPFLGTAGVAGEFPTA | 1 | 106 | 123 |  |

# References

1. von Bergen, M., et al., *Assembly of tau protein into Alzheimer paired helical filaments depends on a local sequence motif ((306)VQIVYK(311)) forming beta structure.* Proc Natl Acad Sci U S A, 2000. **97**(10): p. 5129-5134.

2. Wood, S.J., et al., *Prolines and Amyloidogenicity in Fragments of the Alzheimers Peptide Beta/A4.* Biochemistry, 1995. **34**(3): p. 724-730.

3. Tjernberg, L., et al., *Charge attraction and beta propensity are necessary for amyloid fibril formation from tetrapeptides.* Journal of Biological Chemistry, 2002. **277**(45): p. 43243-43246.

4. Bodles, A.M., et al., *Toxicity of non-abeta component of Alzheimer's disease amyloid, and N-terminal fragments thereof, correlates to formation of beta-sheet structure and fibrils.* European Journal of Biochemistry, 2000. **267**(8): p. 2186-94.

5. Trinh, C.H., et al., *Crystal structure of monomeric human beta-2-microglobulin reveals clues to its amyloidogenic properties.* Proc Natl Acad Sci U S A, 2002. **99**(15): p. 9771-9776.

6. Jones, S., et al., *Amyloid-forming peptides from beta(2)-microglobulin - Insights into the mechanism of fibril formation in vitro.* J Mol Biol, 2003. **325**(2): p. 249-257.

7. Fernandez-Escamilla, A.M., et al., *Prediction of sequence-dependent and mutational effects on the aggregation of peptides and proteins.* Nature Biotechnology, 2004. **22**(10): p. 1302-1306.

8. Padmanabhan, S., M.A. Jimenez, and M. Rico, *Folding propensities of synthetic peptide fragments covering the entire sequence of phage 434 Cro protein.* Protein Science, 1999. **8**(8): p. 1675-1688.

9. Reymond, M.T., et al., *Folding propensities of peptide fragments of myoglobin.* Protein Science, 1997. **6**(3): p. 706-716.

10. Dyson, H.J., et al., *Folding of Peptide-Fragments Comprising the Complete Sequence of Proteins - Models for Initiation of Protein Folding .1. Myohemerythrin.* J Mol Biol, 1992. **226**(3): p. 795-817.

11. Dyson, H.J., et al., *Folding of Peptide-Fragments Comprising the Complete Sequence of Proteins - Models for Initiation of Protein Folding .2. Plastocyanin.* J Mol Biol, 1992. **226**(3): p. 819-835.

12. Kemmink, J. and T.E. Creighton, *Local Conformations of Peptides Representing the Entire Sequence of Bovine Pancreatic Trypsin-Inhibitor and Their Roles in Folding.* J Mol Biol, 1993. **234**(3): p. 861-878.

13. Luisi, D.L., W.J. Wu, and D.P. Raleigh, *Conformational analysis of a set of peptides corresponding to the entire primary sequence of the n-terminal domain of the ribosomal protein L9: Evidence for stable native-like secondary structure in the unfolded state.* J Mol Biol, 1999. **287**(2): p. 395-407.

14. Dragani, B., et al., *Conformational properties of five peptides corresponding to the entire sequence of glutathione transferase domain II.* Archives of Biochemistry and Biophysics, 2001. **389**(1): p. 15-21.

15. Viguera, A.R., et al., *Conformational analysis of peptides corresponding to beta-hairpins and a beta-sheet that represent the entire sequence of the alpha-spectrin SH3 domain.* J Mol Biol, 1996. **255**(3): p. 507-521.

16. Villegas, V., et al., *Protein engineering as a strategy to avoid formation of amyloid fibrils.* Protein Science, 2000. **9**: p. 1700 - 1708.

17. Munoz, V., F.J. Blanco, and L. Serrano, *The Distribution of Alpha-Helix Propensity Along the Polypeptide-Chain Is Not Conserved in Proteins from the Same Family.* Protein Science, 1995. **4**(8): p. 1577-1586.

18. Munoz, V., et al., *Structural-Analysis of Peptides Encompassing All Alpha-Helices of 3 Alpha/Beta-Parallel Proteins - Che-Y, Flavodoxin and P21-Ras - Implications for Alpha-Helix Stability and the Folding of Alpha/Beta-Parallel Proteins.* J Mol Biol, 1995. **247**(4): p. 648-669.

19. RamirezAlvarado, M., L. Serrano, and F.J. Blanco, *Conformational analysis of peptides corresponding to all the secondary structure elements of protein L B1 domain: Secondary structure propensities are not conserved in proteins with the same fold.* Protein Science, 1997. **6**(1): p. 162-174.

20. Blanco, F.J. and L. Serrano, *Folding of Protein-G B1 Domain Studied by the Conformational Characterization of Fragments Comprising Its Secondary Structure Elements.* European Journal of Biochemistry, 1995. **230**(2): p. 634-649.

21. Balbach, J.J., et al., *Amyloid fibril formation by A beta(16-22), a seven-residue fragment of the Alzheimer's beta-amyloid peptide, and structural characterization by solid state NMR.* Biochemistry, 2000. **39**(45): p. 13748-13759.

22. Ivanova, M.I., M.J. Thompson, and D. Eisenberg, *A systematic screen of beta(2)-microglobulin and insulin for amyloid-like segments.* Proc Natl Acad Sci U S A, 2006. **103**(11): p. 4079-4082.

23. Kozhukh, G.V., et al., *Investigation of a peptide responsible for amyloid fibril formation of beta(2)-microglobulin by Achromobacter protease I.* Journal of Biological Chemistry, 2002. **277**(2): p. 1310-1315.

24. Ivanova, M.I., et al., *An amyloid-forming segment of beta 2-microglobulin suggests a molecular model for the fibril.* Proc Natl Acad Sci U S A, 2004. **101**(29): p. 10584-10589.

25. Azriel, R. and E. Gazit, *Analysis of the structural and functional elements of the minimal active fragment of islet amyloid polypeptide (IAPP) - An experimental support for the key role of the phenylalanine residue in amyloid formation.* Journal of Biological Chemistry, 2001. **276**(36): p. 34156-34161.

26. Nilsson, M.R. and D.P. Raleigh, *Analysis of amylin cleavage products provides new insights into the amyloidogenic region of human amylin.* J Mol Biol, 1999. **294**(5): p. 1375-1385.

27. Jaikaran, E.T., et al., *Identification of a novel human islet amyloid polypeptide beta-sheet domain and factors influencing fibrillogenesis.* J Mol Biol, 2001. **308**(3): p. 515-25.

28. Kapurniotu, A., A. Schmauder, and K. Tenidis, *Structure-based design and study of non-amyloidogenic, double N-methylated IAPP amyloid core sequences as inhibitors of IAPP amyloid formation and cytotoxicity.* J Mol Biol, 2002. **315**(3): p. 339-350.

29. Mazor, Y., et al., *Identification and characterization of a novel molecular-recognition and self-assembly domain within the islet amyloid polypeptide.* J Mol Biol, 2002. **322**(5): p. 1013-1024.

30. Koscielska-Kasprzak, K. and J. Otlewski, *Amyloid-forming peptides selected proteolytically from phage display library.* Protein Science, 2003. **12**(8): p. 1675-1685.

31. Giasson, B.I., et al., *A hydrophobic stretch of 12 amino acid residues in the middle of alpha-synuclein is essential for filament assembly.* Journal of Biological Chemistry, 2001. **276**(4): p. 2380-2386.

32. Gustavsson, A., U. Engstrom, and P. Westermark, *Normal Transthyretin and Synthetic Transthyretin Fragments Form Amyloid-Like Fibrils Invitro.* Biochemical and Biophysical Research Communications, 1991. **175**(3): p. 1159-1164.

33. Thompson, M.J., et al., *The 3D profile method for identifying fibril-forming segments of proteins.* Proc Natl Acad Sci U S A, 2006. **103**(11): p. 4074-4078.

34. Otzen, D.E., O. Kristensen, and M. Oliveberg, *Designed protein tetramer zipped together with a hydrophobic Alzheimer homology: A structural clue to amyloid assembly.* Proc Natl Acad Sci U S A, 2000. **97**(18): p. 9907-9912.

35. Maury, C.P.J., et al., *Danish type gelsolin related amyloidosis: 654G-T mutation is associated with a disease pathogenetically and clinically similar to that caused by the 654G-A mutation (familial amyloidosis of the Finnish type).* Journal of Clinical Pathology, 2000. **53**(2): p. 95-99.

36. Nilsson, M.R. and C.M. Dobson, *In vitro characterization of lactoferrin aggregation and amyloid formation.* Biochemistry, 2003. **42**(2): p. 375-382.

37. Haggqvist, B., et al., *Medin: An integral fragment of aortic smooth muscle cell-produced lactadherin forms the most common human amyloid.* Proc Natl Acad Sci U S A, 1999. **96**(15): p. 8669-8674.

38. Westermark, G.T., U. Engstrom, and P. Westermark, *The N-Terminal Segment of Protein Aa Determines Its Fibrillogenic Property.* Biochemical and Biophysical Research Communications, 1992. **182**(1): p. 27-33.

39. Gasset, M., et al., *Predicted Alpha-Helical Regions of the Prion Protein When Synthesized as Peptides Form Amyloid.* Proc Natl Acad Sci U S A, 1992. **89**(22): p. 10940-10944.

40. Patino, M.M., et al., *Support for the prion hypothesis for inheritance of a phenotypic trait in yeast.* Science, 1996. **273**(5275): p. 622-6.

41. Balbirnie, M., R. Grothe, and D.S. Eisenberg, *An amyloid-forming peptide from the yeast prion Sup35 reveals a dehydrated beta-sheet structure for amyloid.* Proc Natl Acad Sci U S A, 2001. **98**(5): p. 2375-2380.

42. Cottingham, M.G., M.S. Hollinshead, and D.J.T. Vaux, *Amyloid fibril formation by a synthetic peptide from a region of human acetylcholinesterase that is homologous to the Alzheimer's amyloid-beta peptide.* Biochemistry, 2002. **41**(46): p. 13539-13547.

43. Vidal, R., et al., *A decamer duplication in the 3 ' region of the BRI gene originates an amyloid peptide that is associated with dementia in a Danish kindred.* Proc Natl Acad Sci U S A, 2000. **97**(9): p. 4920-4925.

44. Orpiszewski, J. and M.D. Benson, *Induction of beta-sheet structure in amyloidogenic peptides by neutralization of aspartate: A model for amyloid nucleation.* J Mol Biol, 1999. **289**(2): p. 413-428.

45. Fandrich, M., et al., *Myoglobin forms amyloid fibrils by association of unfolded polypeptide segments.* Proc Natl Acad Sci U S A, 2003. **100**(26): p. 15463-15468.

46. Baumann, M.H., et al., *C-terminal fragments of alpha- and beta-tubulin form amyloid fibrils in vitro and associate with amyloid deposits of familiar cerebral amyloid angiopathy, British type.* Biochemical and Biophysical Research Communications, 1996. **219**(1): p. 238-242.

47. Iconomidou, V.A., G. Vriend, and S.J. Hamodrakas, *Amyloids protect the silkmoth oocyte and embryo.* Febs Letters, 2000. **479**(3): p. 141-145.

48. Iconomidou, V.A., et al., *Amyloid-like fibrils from an 18-residue peptide analogue of a part of the central domain of the B-family of silkmoth chorion proteins.* Febs Letters, 2001. **499**(3): p. 268-273.
