# Supplementary material for: Identification of properties important to protein aggregation using feature selection
Source: BMC Bioinformatics. 2013 Oct 28;14:314. doi: 10.1186/1471-2105-14-314 (PMC3819749; doi:10.1186/1471-2105-14-314)
Supplement: Additional file 3 — 16 additional features collected from published literatures. [file 1471-2105-14-314-S3.doc]

## Supplementary File 3: 16 additional features collected from published literatures

| **Index ID** | **Description** | **Ref** |
| --- | --- | --- |
| X17196033 | Mean Observed Packing Density for 20 Amino Acid Residues |  |
| X17324296 | Relative experimental aggregation propensities of the 20  natural amino acids used by AGGRESCAN |  |
| X15925383 | Amyloid aggregation propensities of the 20 naturally occurring amino acids (pH7) used by ZYGGREGATOR |  |
| spatial- aggregation- propensity | solvent accessible area of Fully Exposed Side Chains for  20 Amino Acid Residues |  |
| dipole | Property index developed in PAGE |  |
| ali | Property index developed in PAGE |  |
| beta | Property index developed in PAGE |  |
| hydro | Property index developed in PAGE |  |
| volume | Property index developed in PAGE |  |
| pol | Property index developed in PAGE |  |
| apol | Property index developed in PAGE |  |
| surface | Property index developed in PAGE |  |
| solubility | Property index developed in PAGE |  |
| alphaf | Property index developed in PAGE |  |
| betaf | Property index developed in PAGE |  |
| turnf | Property index developed in PAGE |  |

1. Galzitskaya, O.V., S.O. Garbuzynskiy, and M.Y. Lobanov, *Prediction of amyloidogenic and disordered regions in protein chains.* Plos Computational Biology, 2006. **2**(12): p. 1639-1648.

2. Conchillo-Sole, O., et al., *AGGRESCAN: a server for the prediction and evaluation of "hot spots" of aggregation in polypeptides.* Bmc Bioinformatics, 2007. **8**: p. -.

3. Pawar, A.P., et al., *Prediction of "aggregation-prone" and "aggregation-susceptible" regions in proteins associated with neurodegenerative diseases.* J Mol Biol, 2005. **350**(2): p. 379-392.

4. Chennamsetty, N., et al., *Prediction of Aggregation Prone Regions of Therapeutic Proteins.* Journal of Physical Chemistry B, 2010. **114**(19): p. 6614-6624.

5. Tartaglia, G.G., et al., *Prediction of aggregation rate and aggregation-prone segments in polypeptide sequences.* Protein Science, 2005. **14**(10): p. 2723-2734.
